# Antioxidant activity of selected plants extract for palm oil stability via accelerated and deep frying study

**DOI:** 10.1016/j.heliyon.2023.e17980

**Published:** 2023-07-07

**Authors:** Welela Meka Kedir, Abiyot Kelecha Geletu, Getabalew Shifera Weldegirum, Milkesa Fanta Sima

**Affiliations:** Department of Chemistry, College of Natural and Computational Science, Mattu University, Mattu, Oromia, Ethiopia

**Keywords:** Frying oil, Physicochemical, Antioxidant, Lepidium sativum, Aframomum corrorima

## Abstract

Antioxidants are organic compounds that help to prevent lipid oxidation and improve the shelf-life of edible oils and fats. Currently, synthetic antioxidants were used as oil stabilizing agent. However, synthetic antioxidants have been causing various health risks. As a result, natural antioxidants such as most parts of olive plant, green tea, sesame, medicinal plants were plays an important role to retard lipid oxidation. The palm oil was continuously frying at 180 °C for 6 days using *Lepidium sativum* (0.2%w/v) and *Aframomum corrorima* (0.3%w/v) seeds extracts as antioxidant. The physicochemical properties of oil in the herbal extract additive group significantly maintained the oil quality during frying compared to the normal control and the food sample containing group. The L. *sativum* extract had a greater oil stability compared to *A. corrorima* extract. However, the frying oil without herbal extract significantly increase the physicochemical properties of oil such as iodine value, acid value, free fatty acid, total polar compounds, density, moisture content, pH etc. during repetitive frying. The antioxidant activity of the plant extract was outstanding, with an IC_50_ value in the range of 75–149.9 μg/mL when compared to the standard butyl hydroxy anisole, which had an IC_50_ value in the range of 74.9 ± 0.06–96.7 ± 0.75 μg/mL. The total phenolic and flavonoid content of the extract for L. sativum was 128.6 ± 0.00 mg GAE/g, 127.0 ± 0.00 mg QE/g, and 130.16 ± 0.001 mg GAE/g, 105.76 ± 0.02 mg QE/g, respectively. The significant effect of the plant extract on the degradation of oil and the formation of free fatty acids was confirmed by Fourier transform infrared spectroscopy. The result of these study revealed that the ethanolic crude extract of *L. sativum* and *A. corrorima* had a potential natural antioxidant to prevent the degradation of palm oil.

## 1 Introduction

Lipid oxidation is a major cause of degradation during the storage and processing of edible fats, oils, and fat-containing products. It modifies critical quality control standards for fats and oils [1]. It also causes a variety of physical and chemical changes, resulting in significant decomposition [2,3]. Oxidation is a principal cause of quality deterioration, promotes rancidity and food degradation [4]. Oxidation reactions generate free radicals that set off chain reactions [3,5]. Furthermore, oxidative stress causes several fatal diseases in humans, such as cancer. Frying is a popular and long-standing culinary technique used to prepare meals all around the world [2,5]. The frying process degrades oils, causes them to lose their nutritional content, and produces toxic chemicals that are harmful to one's health [6,7]. In order to retard oil oxidation, the use of antioxidants is common in the oil processing industry. Antioxidants are components that prevent the auto-oxidation of oils and fats [8].

Thermal stability after heat processing is one of the most important requirements for a suitable antioxidant in oils and fats [9]. The addition of antioxidants helps delay lipid oxidation and improves the shelf-life of edible oils and fats [10]. Various study reported that synthetic antioxidants such as butylated hydroxytoluene, butylated hydroxyanisole and tert-butyl-hydroquinone, were used as oil stabilizing agent [5]. However, synthetic antioxidants have been linked to health risks, and some have been removed and prohibited in a number of countries. As a result, natural antioxidants such as oleoresins, plant extracts, and volatile oils have received a lot of attention in the field of lipid oxidation [3,5]. Natural antioxidants such as most parts of olive plant, green tea, sesame, medicinal plants were plays an important role to retard lipid oxidation [11]. *Lepidium sativum* has been used in traditional and folklore medicine for the treatment of bronchial asthma, diabetes, and local and rheumatic pain, and the ethanolic extract of the plant has high potential *in vitro* antioxidant activity [12,13].

Natural antioxidants like tocopherol and carotenoids are found in *L. sativum* seed extract, which prevent oil rancidity [14]. Furthermore, around 17 chemical compounds were detected using LC-MS from the seed of *L. sativum* including the important secondary metabolites quercetin and kaempferol, both of which have well-known antioxidant potential [15]. The seed of *L. sativum* contains antioxidant compounds such as 7,10-Hexadecadienoic acid, 11-octadecenoic acid, 7,10,13-hexadecatrienoic acid, and behenic acid [16]. These compounds have a high capacity for free radical scavenging activity. Moreover, the most abundant components in the seeds of *L. sativum* are 1-isocyano-2-methylbenzene (71.63%), benzaldehyde (11.21%), and those compounds are utilized as antioxidants and food preservatives [14]. *Aframomum corrorima* seed is traditionally used as a tonic, laxative, carminative, and purgative, and it is added to food as a preservative [17,18]. *A. corrorima* used as spice and a good source of antimicrobial and antioxidants activity [17]. The predominant chemical compounds discovered in seed oil were 1, 8-cineole (39.3%), sabinene (10.4%), and geraniol (6.8%). The seeds included higher quantities of monoterpenes, such as 1, 8-cineole, sabinene, β-pinene, and geraniol, which accounted for 94% of the total detected compounds, and (E)-nerolidol (4.5%), which is a highly stable oil ingredient [19]. Furthermore, the seed of *A. danielli*, which is related to *A. corrorima*, contained primarily 1, 8-cineole and β-pinene predominated in the leaf, stem, rhizome, and pod oils. In the DPPH assay, the seed oil showed the greatest antioxidant effect [20]. Currently, the oil most frequently used for frying is palm oil, which is the second-largest source of oil in the world, behind soybean oil (FEDIOL, 2012). Due to its high saturation level, it exhibits good oxidative stability. This is the rationale behind the current study's use of palm oil as the frying medium. According to the literature, the suitable temperatures for frying are around 150 °C and only a few studies reported using plant extracts to slow down the oxidation of oils at high temperatures [9].

In this study, Sambussa (made from wheat flora and lentils) was deep-fried in batches at 180 °C in palm oil enriched with *L. sativum* and *A. corrorima* seed extract to investigate the thermal stability. Moreover, essential oil and active components have been used as natural antioxidants for stabilizing the oils [8]. However, essential oils generally have weak antioxidative effects at higher temperatures, whereas they are very effective at storage temperatures due to the volatility of the essential oil. The majority of earlier investigations have concentrated on the essential oils of various plants. To the best of our knowledge, there is no study report describing the use of those plant extracts as oil stabilizing agents. These experiments showed that the best method for stabilizing the oils is to use plant extracts rather than introduce the plants directly. Moreover, the selected plants have well-known antioxidant activity and are accessible at the sample collection site. Therefore, the overall goals of this study were to improve the quality, stability, and shelf life of deep-frying oil using *L. sativum* and *A. corrorima* seed extracts as natural antioxidants.

## 2 Materials and methods

### 2.1 Chemicals and instrument

All the chemicals and reagents used in this study were of analytical grade. FTIR (IFS120 M, Bruker Optik GmbH, Germany) and UV–Visible (LAMBD, double-beam UV/Vis, Germany) spectroscopy were adopted to characterize the oil and plant extracts.

### 2.2 Sample collection and preparation

The seeds of *L. sativum* and *A. corrorima* were collected in Illubabor Zone, Oromia regional state, South Western Ethiopia, in October 2021. The plant samples were identified by botanists, and voucher specimens ET-01 for *L. sativum* and WK-280 for *A. corrorima* were deposited at Department of Biology, College of Natural and Computational Science, Mattu University Herbarium, Mattu, Ethiopia. The common oil type (palm oil) was purchased from Mattu local market and then stored in a refrigerator at −20 °C until used. The food sample (Sambussa) which was made from prepared with wheat flour and lentils were chosen as the food sample since it is a common meal in the study area. The collected seeds of *L. sativum* and *A. corrorima* were washed and air dried under shade. The dried plant seeds were powdered to facilitate the penetration of the extraction solvent into the cell.

### 2.3 Extraction and preliminary physicochemical screening

The powder of each plant (10 g) was soaked with 250 mL of hexane, chloroform, acetone, and ethanol for 24 h using a maceration technique. The qualitative phytochemical screening of the crude extracts of the seeds of *L. sativum and A. corrorima* was evaluated to identify the presence of various phytochemicals such as saponin, alkaloids, steroids, tannins, flavonoids, phenols, terpenoids, glycosides, and quinones with some modification [21,22]. All tests were done in triplicate. Based on the physicochemical analysis result ethanol was selected for further extraction. The powdered plant materials (100 g for each) were soaked with ethanol (1L) for 24 h at room temperature using maceration technique. The solvent extracts were filtered and concentrated using a rotary evaporator at a temperature of 60 °C with a speed of 90 rpm to have a solid consistency and dried at room temperature. Finally, the crude extract was packed in air-tight glass bottles with proper labels and kept in a refrigerator at 4 °C until used for the next experiment [23]. The qualitative phytochemical screening of the plant crude extracts such as saponin, alkaloids, steroid, tannins, flavonoids, phenolic, terpenoids, glycosides and quinones were investigated using a standard methods reported from the previous similar study [14].

### 2.4 Antioxidant activity of Lepidium sativum and Aframomum corrorima seed extract

#### 2.4.1 DPPH assay

The DPPH radical-scavenging activity of plant extracts was determined by adding various concentrations of test extracts to 2.9 mL of a 0.004% (w/v) ethanol solution of DPPH. After 30 min of incubation at room temperature, the absorbance was measured at 517 nm against a blank [24]. The IC_50_ values (concentration of sample required to scavenge 50% of free radicals) were calculated from the regression equation. Butylated hydroxy anisole (BHA) was used as a positive control, and all tests were performed in triplicate. DPPH's free radical inhibition (I%) was calculated using equation (1).

(1) $$I\%=\frac{A_{blank-}Asample}{Ablank}x\;100$$

Where A is Absorbance

#### 2.4.2 Ferric ions (Fe^3+^) reducing antioxidant power assay

The reducing power assay was performed using the method described by previous similar study report with minor modifications [25]. An aliquot of 0.2 mL of various concentrations of the extracts (25–125 μg/mL) were mixed separately with 0.5 mL of phosphate buffer (0.2 M, pH 6.6) and 0.5 mL of 1% potassium ferricyanide. The mixture was incubated in a water bath at 50 °C for 20 min. After cooling to room temperature, 0.5 mL of 10% trichloroacetic acid was added, followed by centrifugation (769.23g) for 10 min. The supernatant (0.5 mL) was collected and mixed with 0.5 mL of distilled water. Ferric chloride (0.1 mL of 0.1%) was added to it, and the mixture was left at room temperature for 10 min. The absorbance was measured at 700 nm, and BHA was used as a positive control. The ability of the extracts to reduce Fe^3+^ to Fe^2+^ was calculated using the following equation (2):

(2) $$Reducing\;power\;assay\;\left(\%\right)=\frac{Acontrol_{-}Asample}{Asample}x\;100$$

Where A is Absorbance

#### 2.4.3 Hydrogen peroxide scavenging assay

The extract's ability to scavenge hydrogen peroxide (H_2_O_2_) was determined using similar method from the previous report with a slight modified [26]. An aliquot of 0.1 mL of extracts (25–125 μg/mL) was transferred into the Eppendorf tubes, and their volume was made up to 0.4 mL with 50 mM phosphate buffer (pH 7.4) followed by the addition of 0.6 mL of H_2_O_2_ solution (2 mM). The reaction mixture was vortexed, and after 10 min of reaction time, its absorbance was measured at 230 nm. BHA was used as the positive control and the ability of the extracts to scavenge the H_2_O_2_ was calculated using the following equation (3):

(3) $$Percentage\;of\;H_{₂}O₂\;scavenging\;activity\;\%=\frac{Acontrol_{-}Asample}{Asample}x\;100$$

Where A is Absorbance

#### 2.4.4 Phosphomolybdenum assay

The phosphorus molybdenum assay was conducted by preparing an aliquot of 0.1 mL of sample solution of different concentrations (25–125 μg/mL) treated with 1 mL of reagent solution (0.6 M sulfuric acid, 28 mM sodium phosphate and 4 mM ammonium molybdate) [27]. The tubes were incubated at 95 °C in a water bath for 90 min. The samples were cooled to room temperature and their absorbance was recorded at 765 nm. BHA was used as the positive control. BHA was used as the positive control and the scavenging ability of the extracts was calculated using the following equation (4):

(4) $$Antioxidant\;activity\;\%=\frac{Acontrol_{-}Asample}{Asample}x\;100$$

Where A is Absorbance

#### 2.4.5 Assay for total phenolics

**Fig. 1:**
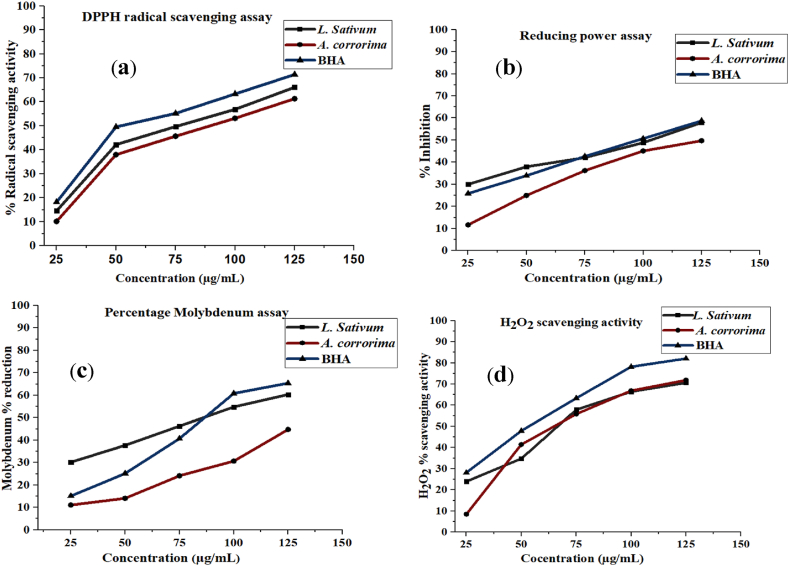
Antioxidant activity of L. sativum and A. corrorima seed extract compared with the positive control BHA.

The total phenolic content (TPC) was determined using the Folin-Ciocalteu reagent [28]. Briefly, 0.01 g of the crude extract was dissolved in 10 mL of ethanol and vortexed until the mixture became a homogenous stock solution. For the stock solution, 0.2 mL of the supernatant was mixed with 0.8 mL of distilled water. 0.1 mL of Folin-Ciocalteu reagent was added and left for 3 min at room temperature. Then, 0.8 mL of 20% (w/v) Na_2_CO_3_ was added into the mixture and incubated for 2 h in the dark. The absorbance was measured using a Uv–Vis spectrophotometer at 765 nm. Gallic acid was used as a standard, and the absorbance, y, obtained after analysis for each plant sample was used in the equation y = 0.0098x – 0.2228, R^2^ = 0.9991 where x is standard concentration (Fig. 1A in the attached supplementary material). Then, the value obtained, x, was substituted in C1 in equation C=C1 x V/m, where C = is the total phenolic content in mg GAE/g, C1 = is the concentration of gallic acid established from the standard curve, and V is the volume and m is the mass of the extract used.

#### 2.4.6 Assay for total flavonoid

The total flavonoid content (TFC) was assessed by the aluminum chloride colorimetric method [29]. Briefly, 0.01 g of the crude extract was dissolved in 10 mL of ethanol and vortexed until it became a homogenous stock solution. 0.2 mL of the extract supernatant was mixed with 0.15 mL of 5% NaNO_2_ and incubated in the dark for 6 min at room temperature. Then, 0.15 mL of 10% (w/v) AlCl_3_ was added to the mixture and kept in the dark for 6 min at room temperature. After that, 0.8 mL of 10% (w/v) NaOH was added into the mixture and incubated in the dark for 15 min at room temperature. The absorbance was measured using a Uv–Vis spectrophotometer at 510 nm. Quercetin (80% (v/v) ethanol) was used as a standard, and the absorbance, y, obtained after analysis for the plant sample was used in the equation y = 0.066x – 0.0142, R^2^ = 0.9991 obtained from the standard curve (Fig. 1A in the in the attached supplementary material supplementary material).

### 2.5 Accelerated oxidation study

The collected oil sample was heated to 60 °C before the extract was added, and then 0.1%, 0.2%, 0.3%, and 0.4% were added and mixed to make sure they were thoroughly dissolved [30]. For the comparison BHA and samples without any antioxidants were used as the positive and negative controls, respectively. The group is classified as Normal (G-1), BHA-0.02% (G-2), LS-0.1% (G-3), LS-0.2% (G-4), LS-0.3% (G-5), LS-0.4% (G-6), AC-0.1% (G-7), AC-0.2% (G-8), AC-0.3% (G-9), and AC-0.4% (G-10). All samples were heated at 180 °C for 0, 12, 24, 48, and 72 h. Samples were collected, cooled to 60 °C, flushed with nitrogen, and then kept at −20 °C before analysis. The physicochemical analysis results of the oil, such as SV, AV, PV, IV, TPC, CD, and CT for the optimization of the plant concentration, were reported and attached as supplementary material in Fig. 3, Fig. 4A as a reference.

### 2.6 Deep frying protocol

The oil was fried for 6 h per day for a total of 6 days. Deep frying was carried out in a stainless steel electrical open fryer (10 L oil capacity) [31]. The treatments were conducted simultaneously in Group-I (oil without any additives), Group-II (normal oil with 0.02% BHA), Group-III (normal oil with 0.2% w/v *L. sativum* extract and food), Group-IV (normal oil with 0.3% w/v *A. corrorima* extract and food), and Group-V (normal oil with food). A sample before frying was taken to represent the sample for day 0. The remaining oil was heated to 180 ± 2 °C and allowed to equilibrate at this temperature for 30 min. About 14 batches of 80g food were fried for 2.5 min per day at 30 min intervals for 6 h. Approximately 100 mL of oil samples were collected from each fryer and introduced into amber bottles at the end of each day. All oil samples were flushed with slow bubbles of nitrogen from the bottom of the bottles and stored at −20 °C prior to physical and chemical analysis. Rapid measurements were taken while there was no moisture (bubbles) in the frying oil after each cycle. The effect of repetitive frying of oil samples on the physicochemical parameter was evaluated [32].

### 2.7 Physicochemical and quality assessment of deep frying oils

The physicochemical characteristics of deep-frying oil in each cycle were investigated. The physicochemical properties, such as acid value, refractive index, iodine value, saponification number, moisture content, and pH, were analyzed by the standard protocol of oil analysis, the AOAC official method (969.17). Several methods for the determination of the quality of deep-frying oils have been developed based on physical and chemical parameters. The oxidation parameters such as free fatty acids (FFAs), peroxide value (PV), iodine value (IV), conjugated dienes (CD), and conjugated trienes (CT) were the major parameters to determine the frying oil deterioration [33]. The density and anisidine value of the oil were determined by the method reported in the previous literature [34,35]. The conventional analytical methods, including titrimetric and spectrophotometric techniques, were adopted to overcome the physicochemical analysis result following the guidelines of the official methods of the American Oil Chemists’ Society (1998) [33,36].

### 2.8 Statistical analysis

The commercial statistical packages (SPSS, version 25) were used to the plant extracts antioxidant activities and physicochemical parameters of oil were performed in triplicates. The statistical differences and homogeneity among the groups were verified using one way ANOVA. The normality of the data were verified using Kurtosis statistics test. The analysis of variance for individual parameters was performed using a Tukey post hoc test to identify the statistical difference on each groups differ from other on the basis of mean values to analyze the significance of multiple comparison measurement of the data at confidence level of 95% (p < 0.05).

## 3 Result

### 3.1 Percentage yield of the crude extract and phytochemical analysis

The percentage yields of *L. sativum* crude extracts were 8%, 17%, 30%, and 41% for hexane, chloroform, acetone, and ethanol, respectively. Moreover, the percentage yields of *A. corrorima* extract were 6% (hexane), 13% (chloroform), 19% (acetone), and 28.5% (ethanol), respectively. This study showed that hexane, chloroform, acetone, and ethanolic extracts of the seeds of *L. Sativum* and *A. corrorima* have a range of secondary metabolites such as saponin, steroids, phenol, terpenoids, flavonoids, quinones, alkaloids, glycosides, and tannins (Table 1).

**Table 1:** Preliminary phytochemical screening of *L. sativum* and *A. corrorima* crude extract.

No	Phytochemicals	Test Methods	Crude extracts							
			L. sativum				*A.* corrorima			
			HE	CE	AE	ET	HE	CE	AE	ET
							
1	Alkaloid	Wagner test	-	+	-	++	-	-	+	+
2	Phenol	FCl_3_test	-	+	+	++	-	+	++	++
3	Flavonoids	NaOH test	-	++	++	++	-	++	+	++
4	Saponin	Foam test	+	-	+	-	+	+	+	++
5	Terpenoids	Salkowski test	+	++	++	-	-	++	-	+
6	Quinone	HCl test	-	+	-	+	-	+	-	-
7	Steroid	Liebermann's Burchard test	+	+	-	-	-	+	-	+
8	Glycoside	NaOH test	-	-	+	++	-	-	+	++
9	Tannin	Braymer's test	+	+	++	++	+	+	++	++

**Note**: HE: hexane, CE: chloroform, AE: acetone, ET: ethanol, - = absence, +: presence, ++ = present in high concentration.

### 3.2 Total phenolics and flavonoid content

**Fig. 2:**
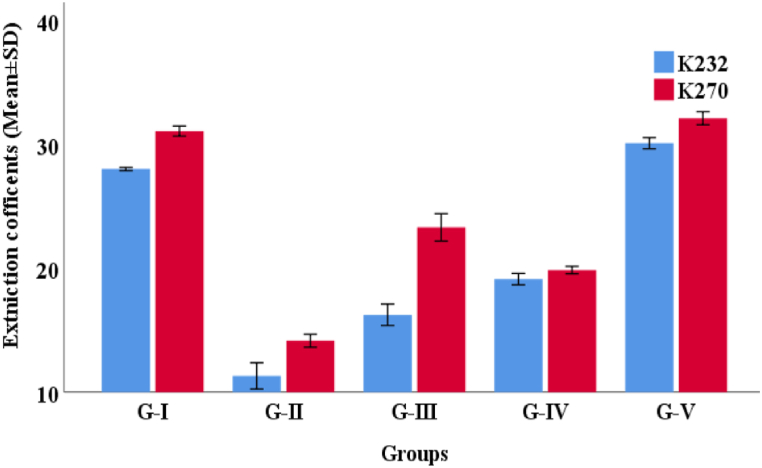
The conjugate diene and conjugate triene of the frying oil. Note: K: Extinction coefficient at 232 and 270 nm.

The TPC of the ethanolic seed extracts of *L. sativum* and *A. corrorima* were 128.6 ± 0.00 and 127.0 ± 0.00 mg GAE/g gallic acid equivalents, respectively (Table 2 and Fig. 1A in the attached supplementary material). The TFC of the crude ethanolic seed extract is expressed as mg quercetin equivalent (QE) per gram dry extract weight (Table 2 and Fig. 2A in the attached supplementary material). There was a significant amount of total flavonoid content (TFC) in both *L. sativum* and *A. corrorima*, which were estimated to be 130.16 ± 0.01 and 105.76 ± 0.02 mg QE/g, respectively.

**Table 2:** Total phenolic, total flavonoid content and IC_50_ values of the plant extract.

Ethanolic Plant extract	mg GAE/g	mg QE/g		IC_50_ (μg/mL)		
	TPC	TFC	DPPH	RPA	PMA	H_2_O_2_
*L. sativum*	128.6 ± 0.00	130.16 ± 0.001	84.7 ± 0.02	97.2 ± 0.57	89.4 ± 0.75	75.9 ± 0.31
*A. corrorima*	127.0 ± 0.00	105.76 ± 0.02	92.74 ± 0.02	116.1 ± 0.28	149.9 ± 0.70	77.3 ± 0.58
BHA	–	–	74.9 ± 0.06	96.7 ± 0.75	83.1 ± 0.84	83.4 ± 0.26

**Note**: RPA = reducing power assay, PMA=Phosphomolybdenum assay.

**Fig. 3:**
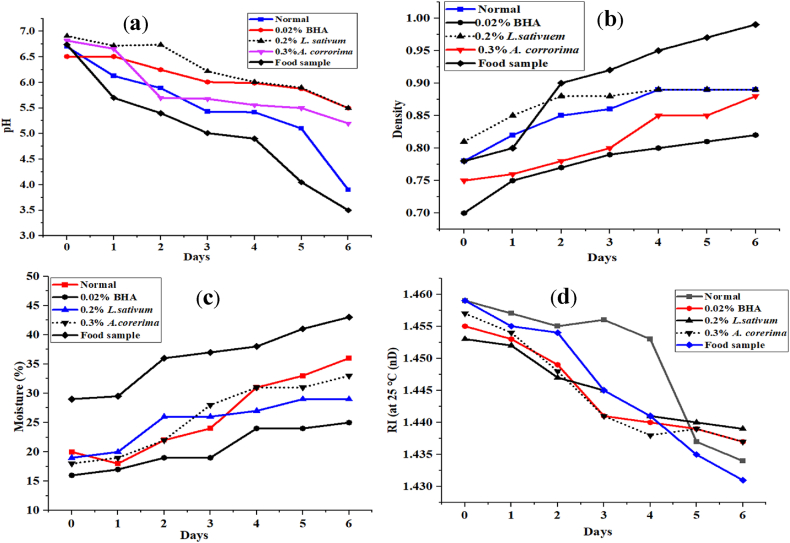
The moisture content, pH, density and refractive index of oil during deep frying.

**Fig. 4:**
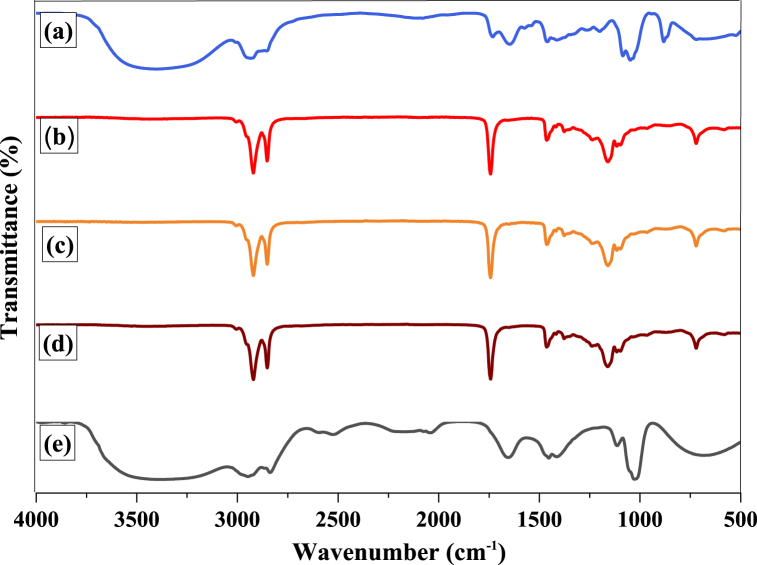
Fourier transforms infrared spectroscopy of frying oil. *(****Note****: a=normal control, b=Positive control, c=0.2% L. sativum, d=0.3% A. corrorima, e = food sample containing group).*

### 3.3 Antioxidant activity

The antioxidant activity of the plant extracts was evaluated using the DPPH free radical scavenging activity assay, hydrogen peroxide inhibition assay, phosphor-molybdenum assay, and ferric reducing power assay (Table 2). The inhibitory concentration (IC_50_) of the different assays was carried out using the regression equation. A lower IC_50_ value indicates a greater potential to scavenge free radicals. The IC_50_ value of BHA (the positive control) was lower than the corresponding plant extract. However, the IC_50_ values of L. sativum (75.9 ± 0.31 μg/mL) and A. corrorima extract (77.3 ± 0.58 μg/mL), which were lower than BHA (83.4 ± 0.26 μg/mL) at the hydrogen peroxide scavenging assay, indicated that the plant extract had stronger antioxidant activity compared to BHA.

#### 3.3.1 Free radical scavenging activity

The percentage free radical scavenging activity of ethanolic extracts of *L. sativum* and *A. corrorima* increased with increasing concentration (25–125 g/mL). For the scavenging activity, the hydrogen-donating ability of the extract toward the DPPH free radical was evaluated. Both plant extracts increase the free radical scavenging activity (%) with increasing concentration (Fig. 1a). The higher scavenging activity was recorded on the ethanolic extract at 125 μg/m for *L. sativum* (66.03 ± 0.774 μg/mL), which is comparable to the positive control BHA (71.38 ± 0.834 μg/mL). Moreover, the DPPH quenching ability of *A. corrorima* increases significantly with increasing concentration (Fig. 1a).

#### 3.3.2 Reducing power assay

The reducing power of the extract was measured for a concentration up to 125 μg/mL and showed a significant increment as the concentration increased (Fig. 1b). Among the two tested plants, *L. sativum* seed extract possessed the highest free radical reducing activity (57.89 ± 0.254 μg/mL) compared to *A. corrorima* (49.68 ± 0.763 μg/mL) at 125 μg/mL. However, when compared to BHA (58.68 ± 0.39 μg/mL), the two plant extracts have a lower ability to reduce free radicals (Fig. 1b).

#### 3.3.3 Phosphomolybdenum assay

The free radical inhibition of plant extracts increases with concentration (Fig. 1c). The antioxidant activity of the *L. sativum* (60.30 ± 0.151 μg/mL) extracts was significantly increased compared to the *A. corrorima* extract (44.72 ± 0.362 μg/mL) at 125 μg/mL. In addition, the molybdenum ion concentration reduction of the ethanolic extract of *L. sativum* showed comparable radical scavenging activity with the positive control BHA (79.39 ± 0.69 μg/mL).

#### 3.3.4 Hydrogen peroxide scavenging assay

The hydrogen peroxide scavenging activity of *L. sativum* and *A. corrorima* seed extracts was investigated within the range of concentrations (25–125 μg/mL) as shown in Fig. 1d. The ethanolic extract of the *L. sativum* and *A. corrorima* seeds (125 μg/mL) displayed a strong percentage H_2_O_2_ scavenging activity (70.60 ± 0.72 μg/mL) and (71.86 ± 0.63 μg/mL) respectively), whereas it was 82.03 ± 0.69 μg/mL for the positive control BHA group.

### 3.4 Accelerated oxidative study

To evaluate the effect of frying during an accelerated oxidative study, different concentrations of plant extract (0.1–0.4%) were investigated and compared with the positive control BHA for 0–72 h. For the optimization of the effect of plant extract, the physicochemical properties of frying oil, such as acid value, saponification value, iodine value, and peroxide value were depicted as supplementary materials in Fig. 3A, respectively. Furthermore, the total polar compound and conjugated diene and conjugated triene levels of frying oil during the accelerated oxidative study were depicted as supplementary material in Fig. 4A. Based on the optimized concentration, L. sativum (0.2%) and A. corrorima (0.3%) showed a comparable protective effect with the positive control BHA. Therefore, these two concentrations of plant extract were selected for the deep-frying protocol as oil stabilizers.

### 3.5 Deep frying study

During the accelerated oxidative study, the deep-frying protocol was followed for six days in a row, and the concentration of plant extract was optimized based on the physicochemical analysis of frying oil at various concentrations. During the optimization process *L. sativum* (0.2% w/v) and *A. corrorima* (0.3% w/v) plant extracts were chosen for the deep-frying study.

#### 3.5.1 Saponification value, acid value and free fatty acid content

The SV, AV, and FFA content of oils during repetitive frying were evaluated and depicted in Table 3. The saponification values of deep-frying oil increased with the increase in the frying cycle. There was no significant difference between the normal controls (Group 1) and all the others. Significant variations in SV between each group during the initial day of frying were recorded. There was a significant difference between the normal control and the plant extract-containing group on day 1 (P < 0.05). However, there is no significant difference between the positive and normal control groups. The SV in group I was statistically significant compared to groups III, IV, and V (P < 0.05), but non-significant compared to the positive control (group II). At day 2, the SV of the normal control group was significantly different compared to groups IV and V (P < 0.05). However, the positive control group was statistically significant compared with groups III, IV, and V. The SV of group V was significantly different compared to the entire group (P < 0.05). Furthermore, the SV of group-I at 3, 4, 5, and 6 days was significantly different compared to the entire group. The acid value (AV) of repetitive frying oil was studied for six continuous days. There was a significant increment in the AV of palm oil during the frying period for the entire group. Initially, the AV of group V (1.30 ± 0.01) was higher than the normal, positive control and the plant extract additive group (P < 0.05). However, after the 1st day of frying, the AV of the normal control group was significantly higher compared to the plant additive group and decreased non-significantly compared to the positive control and food sample-containing group (group V). After one day, the AV of each frying oil was significantly different. Furthermore, significant increases in groups I and V were observed when compared to the positive and plant antioxidant-containing groups. The FFA (%) content of oil in each group increased with the frying period. However, after day 1, the FFA (%) content of the frying oil with food sample was significantly higher than the positive control and the plant antioxidant additive group. However, there was no significant difference between the normal control and group V. After days 1 through 6, the FFA (%) of the normal control group was statistically significant compared to the entire group.

**Table 3:** Change in the quality of oil during repetitive frying.

Parameters	Days	Frying system				
		Normal	BHA (0.02%)	LS (0.2%)	AC (0.3%)	Sambussa
SV	0	158.93 ± 0.06	159.60 ± 1.44	161.30 ± 1.55	159.37 ± 0.55	160.33 ± 0.58
	1	161.01 ± 0.01	160.00 ± 0.00	165.67 ± 1.1	163.40 ± 0.69	170.33 ± 0.58
	2	164.27 ± 0.46	161.67 ± 1.52	166.33 ± 0.58	170.67 ± 1.52	175.73 ± 0.64
	3	177.67 ± 0.58	162.50 ± 0.50	169.67 ± 0.58	173.03 ± 0.04	178.27 ± 0.23
	4	180.05 ± 0.05	165.67 ± 0.58	170.60 ± 1.22	175.60 ± 0.79	189.23 ± 0.21
	5	183.47 ± 0.50	166.83 ± 0.29	171.67 ± 0.58	176.67 ± 1.53	198.43 ± 0.51
	6	194.33 ± 1.08	170.27 ± 0.46	178.97 ± 1.00	180.00 ± 1.00	205.13 ± 1.20
AV	0	0.84 ± 0.05	0.81 ± 0.01	0.79 ± 0.02	0.86 ± 0.06	1.30 ± 0.06
	1	1.79 ± 0.01	1.82 ± 0.02	1.60 ± 0.05	1.72 ± 0.01	1.94 ± 0.01
	2	2.45 ± 0.01	2.23 ± 0.03	2.36 ± 0.01	2.52 ± 0.02	2.94 ± 0.01
	3	3.13 ± 0.01	2.51 ± 0.01	2.52 ± 0.02	2.79 ± 0.01	3.41 ± 0.01
	4	3.31 ± 0.36	2.81 ± 0.02	2.74 ± 0.02	3.10 ± 0.01	4.02 ± 0.01
	5	3.65 ± 0.05	3.03 ± 0.03	2.80 ± 0.06	3.11 ± 0.01	4.30 ± 0.02
	6	4.02 ± 0.02	3.09 ± 0.01	3.12 ± 0.02	3.30 ± 0.02	4.90 ± 0.02
%FFA	0	0.47 ± 0.08	0.50 ± 0.00	0.42 ± 0.03	0.52 ± 0.70	0.51 ± 0.02
	1	0.93 ± 0.06	0.80 ± 0.09	0.73 ± 0.04	0.82 ± 0.02	0.96 ± 0.05
	2	1.23 ± 0.03	1.09 ± 0.02	0.81 ± 0.02	1.27 ± 0.01	1.46 ± 0.03
	3	1.42 ± 0.01	1.17 ± 0.01	1.10 ± 0.01	1.31 ± 0.03	1.93 ± 0.06
	4	1.94 ± 0,02	1.24 ± 0.01	1.23 ± 0.01	1.32 ± 0.05	1.69 ± 0.06
	5	2.88 ± 0.03	2.23 ± 0.01	2.36 ± 0.01	2.42 ± 0.01	3.00 ± 0.02
	6	2.97 ± 0.06	2.41 ± 0.02	2.50 ± 0.02	2.62 ± 0.01	3.03 ± 0.02

**Note**: AC = A. corrorima, LS = L. sativum, SV = saponification value, AV = acid value, FFA = free fatty acid. The experimental data was in triplicate and the statistical value significant at (P < 0.05).

#### 3.5.2 Peroxide value, p-anisidine value and total oxidation

In this study, the PV, *p-AV*, and TOXOX values of frying oil in each group increased throughout the study period (Table 4). There was a significant difference between the normal control group and the entire group during the initial period (P < 0.05). The *p-AV* of oil increases at random as the frying time increases for the entire group. The higher *p-AV* of the oil was observed in groups I and V throughout the frying periods. The PV of frying oil under positive control significantly decreased compared to the other group. After day 1, the PV of frying oil dramatically increases with increased frying time. There was a significant difference observed between the normal control group and the entire group in the initial period (p < 0.05). However, group V had the highest *p-AV* of frying oil from the first (6.06 ± 0.02 to the final (36.22 ± 0.31) day of frying). In this study, the higher peroxide values in the frying oil after 6 days that contained a food sample (Sambussa) (37.00 ± 0.95 meq O_2_/kg) were an indication of the higher degree of oxidation. The TOXOX value results were also compiled and depicted in Table 4. During the initial period of frying, the TOTOX in the normal control group was statistically significant compared to the groups II, III, and V (P < 0.05).

**Table 4:** Quality changes in palm during frying.

Parameters	Days	Frying system				
		Normal	BHA (0.02%)	LS (0.2%)	AC (0.3%)	Sambussa
PV	0	5.80 ± 0.01	4.97 ± 0.06	5.32 ± 0.02	5.98 ± 0.07	7.89 ± 0.01
	1	8.96 ± 0.06	5.93 ± 0.06	6.99 ± 0.01	8.52 ± 0.02	17.05 ± 0.05
	2	12.05 ± 0.05	5.99 ± 0.09	8.97 ± 0.07	9.94 ± 0.09	23.06 ± 0.06
	3	16.30 ± 0.60	7.30 ± 0.44	10.07 ± 0.21	11.83 ± 0.21	24.69 ± 0.27
	4	21.04 ± 0.04	6.89 ± 0.11	13.04 ± 0.05	15.05 ± 0.05	28.03 ± 0.03
	5	25.68 ± 0.59	8.02 ± 0.03	12.57 ± 0.59	16.36 ± 0.55	31.30 ± 0.61
	6	29.33 ± 0.58	8.95 ± 0.04	13.95 ± 0.05	19.67 ± 0.35	37.00 ± 0.95
p-AV	0	5.70 ± 0.08	5.39 ± 0.01	6.00 ± 0.02	5.57 ± 0.01	6.06 ± 0.02
	1	22.12 ± 0.21	5.65 ± 0.05	8.83 ± 0.11	17.65 ± 0.74	26.15 ± 0.18
	2	25.38 ± 0.51	9.32 ± 0.08	13.36 ± 0.37	18.25 ± 0.45	26.22 ± 0.31
	3	29.62 ± 1.16	13.34 ± 0.27	14.17 ± 0.21	20.46 ± 0.36	33.19 ± 0.33
	4	33.07 ± 0.12	15.73 ± 0.06	16.11 ± 0.12	30.18 ± 0.28	34.47 ± 0.56
	5	36.08 ± 0.04	16.49 ± 0.18	18.07 ± 0.08	35.18 ± 0.27	36.22 ± 0.31
	6	39.10 ± 0.36	16.47 ± 0.41	20.36 ± 0.47	36.39 ± 0.30	41.40 ± 0.59
TOTOX	0	17.23 ± 0.15	15.46 ± 0.15	16.62 ± 0.08	17.43 ± 0.15	21.85 ± 0.05
	1	40.39 ± 0.15	17.74 ± 0.15	22.80 ± 0.08	35.54 ± 0.14	60.35 ± 0.57
	2	48.72 ± 0.35	21.32 ± 0.05	30.91 ± 0.14	38.08 ± 0.06	70.79 ± 0.03
	3	54.86 ± 1.80	27.55 ± 0.07	34.33 ± 0.89	44.17 ± 0.94	83.02 ± 1.54
	4	64.43 ± 0.08	29.32 ± 0.05	41.76 ± 0.10	60.07 ± 0.19	90.21 ± 0.43
	5	75.67 ± 0.38	32.13 ± 0.28	44.10 ± 0.34	67.13 ± 0.23	99.39 ± 0.12
	6	99.83 ± 0.60	34.93 ± 0.29	48.90 ± 0.06	74.60 ± 0.06	112.62 ± 0.37

**Note**: AC = A. corrorima, LS = L. sativum, PV = peroxide value, P-AV = anisidine value, TOTOX = total oxidation. The experimental data was in triplicate and the statistical value significant at (P < 0.05).

#### 3.5.3 Total polar compounds (TPc) and iodine value (IV) of frying oil

The TPc of oil in the food sample containing group (group V) was higher than the overall TPc (Table 5). A significant difference was observed between the normal control and the rest of the others (P < 0.05) in the initial and day-1 periods of frying. However, there was no significant difference between the TPc of the plant extract additive and the normal control group (P > 0.05) at day 2. At day 2, there was no significant variation observed between the positive control and the plant extract additive group. However, 0.2% w/v significantly decreases the TPc of frying oil compared to the 0.3% w/v additive group. Moreover, the plant extract additive group significantly decreases the TPc of oil compared to the normal control and frying oil with food (group V). The IV of frying oil decreased throughout the study period. There was no significant difference between the normal, positive control, and plant extract additive groups in the initial day of frying (P > 0.05). However, there is a significant difference between normal cooking and frying oil-containing foods. Similarly, up to day 3 (Table 5), the IV of frying oil in the positive control and the 0.2% w/v L. sativum and 0.3% w/v plant extract additive groups were comparable. However, after day 3, there was a significant difference between the 0.3% w/v A. corrorima additive and the positive control group.

**Table 5:** Change in the quality of oil such as TPc and IV during repetitive frying.

Parameters	Days	Frying system				
		Normal	BHA (0.02%)	LS (0.2%)	AC (0.3%)	Sambussa
TPc	0	10.24 ± 0.13	6.86 ± 0.06	9.06 ± 0.03	8.63 ± 0.45	14.10 ± 0.10
	1	17.26 ± 0.16	10.39 ± 0.01	10.97 ± 0.06	11.60 ± 0.02	16.86 ± 0.06
	2	19.49 ± 0.51	11.13 ± 0.40	11.43 ± 0.52	11.90 ± 0.10	18.81 ± 0.05
	3	23.74 ± 0.07	11.90 ± 0.01	12.53 ± 0.31	12.73 ± 0.15	20.67 ± 0.03
	4	23.86 ± 0.16	12.82 ± 0.02	12.85 ± 0.07	13.02 ± 0.03	25.43 ± 0.08
	5	23.82 ± 0.17	12.80 ± 0.08	12.97 ± 0.15	15.36 ± 0.06	29.25 ± 0.18
	6	27.44 ± 0.06	13.07 ± 0.04	14.97 ± 0.06	16.93 ± 0.06	30.28 ± 0.36
IV	0	62.70 ± 2.14	60.30 ± 0.61	62.73 ± 0.64	60.17 ± 0.15	59.80 ± 0.10
	1	52.67 ± 0.58	60.06 ± 0.06	60.20 ± 0.20	59.50 ± 0.44	49.10 ± 1.01
	2	47.57 ± 0.51	57.23 ± 0.21	57.06 ± 0.05	55.03 ± 0.03	42.04 ± 0.05
	3	44.70 ± 0.61	54.13 ± 0.12	54.83 ± 0.76	53.30 ± 0.26	40.10 ± 0.10
	4	41.10 ± 0.17	55.23 ± 0.21	53.26 ± 0.21	47.63 ± 1.09	38.23 ± 0.21
	5	37.83 ± 0.21	55.16 ± 0.20	53.20 ± 0.27	44.33 ± 0.58	35.00 ± 1.00
	6	36.43 ± 0.38	55.43 ± 0.37	53.40 ± 0.53	43.23 ± 0.21	33.03 ± 0.04

**Note**: AC = A. corrorima, LS = L. sativum the experimental data was in triplicate and the statistical value significant at (P < 0.05).

#### 3.5.4 Conjugate diene and conjugate trienes

The conjugate diene and conjugate triene levels of the frying oil were investigated using UV–visible spectroscopy with respect to the extinction coefficient (K). It can be observed that all samples exhibited a steady increase in absorbance between 232 nm and 270 nm, indicating an increase in the formation of both conjugated dienes and trienes during repeated frying (Fig. 2).

#### 3.5.5 Effect of pH, density and moisture content

The pH, density, moisture content, and refractive index of the oil were evaluated throughout deep frying and are depicted in Fig. 3. The pH of a cooking-quality vegetable oil is normally kept neutral and usually ranges from 6.9 to 6.7 (Fig. 3a). The density of the frying oil was also evaluated during the deep-frying study and is shown in Fig. 3b. Increase the frying cycle to increase its density for all samples. The density of the food sample containing group was greater than the entire group, which was 0.89 ± 0.35. However, in the final period of frying, the density was lower on the positive control (0.82 ± 0.34) and *A. corrorima* plant extract (0.3%) additive group (0.88 ± 0.97) (Fig. 3b). The parentage moisture content of deep-frying oil showed a great deal of variation between the groups throughout the study periods, as depicted in Fig. 3c. The moisture content of food samples containing oil (group V) and the normal control (group I) was higher than that of the plant extract additive group. However, the moisture content of the positive control group was lower than that of the food and plant extract additive group. The greater moisture content of group V at day 6 of frying was 43.09 ± 0.99. The RI value of the oil was investigated, and it was observed to be in the range of 1.432–1.462 throughout the deep-frying study (Fig. 3d).

### 3.6 Fourier transform infrared spectroscopy of oil after 6^th^day of frying

After heating and frying, the level of oxidation was assessed using FTIR spectroscopy. In this study, five samples (one sample for each group) were investigated. The FTIR spectra of frying palm oil showed a significant difference in the band at room temperature (Fig. 4).

## 4 Discussion

In this study the effect of plant extract on the stability of palm oil were evaluated and the palm oil was continuously frying at 180 °C for 6 days. The antioxidant activity, TPC and TFC of *L. sativum* and *A. corrorima* seed extracts were discussed. The antioxidant and oil stability of potential of the plant might be due to the secondary metabolite found in the extract. Secondary metabolites are natural products that are primarily produced by bacteria, fungi, and plants. They are low-molecular-weight molecules with a range of biological importance, including antioxidants and antimicrobial activity [37, 38]. The phytochemical screening result of the plant extract revealed that, the presence various secondary metabolite that helps to stabilize oil during frying. The ethanolic extract of *L. sativum* showed a better solvent to extract various secondary metabolite compared to other solvent extracts. This study confirmed the previous study's findings [37] and stated that *L. sativum* was rich in alkaloids, glycosides, phenols, terpenoids, flavonoids, and other secondary metabolites. Similarly, the ethanolic crude extract of *A. corrorima* showed a positive result for phenol, flavonoid, tannin, and glycoside. Secondary metabolites, particularly phenol and flavonoids, have been shown to have significant radical scavenging activity [38]. The antioxidant activity of the plant extract might be due to the bioactivity potential of the secondary metabolite [14]. Flavonoids, which are phenolic compounds present in medicinal plants, exhibit antioxidant activity [39].

The phytochemicals such as alkaloids, flavonoids, and terpenes are essential in antioxidant, analgesic, neuroprotective, antimicrobial, and antimalarial actions [14, 38]. They also serve as anticancer and antidiabetic agents [24]. In general, the phytochemical screening results of the plant seed extracts might have promising medicinal applications since tannin; terpenoids, saponins, phenols, and flavonoids are among the major phytochemicals of the plant seed extracts [14]. The successful screening of phenolic and flavonoid compounds can be influenced by a number of factors, including sample size, storage conditions, weather, extraction method, the presence of any interfering substances, and the solvent [38,39]. However, no single solvent or mixture of solvents has been shown to effectively extract phenolic compounds from these two species.

The phenolic hydroxyl groups have a remarkable ability to scavenge free radicals [28]. On the other hand, flavonoids are biologically important compounds with a broad spectrum of biological activities such as antioxidant, anticancer, anti-inflammatory, anti-allergic, anti-angiogenic, and anti-allergic. The TPC of the methanolic and ethanolic extracts of *L. sativum* were 94.48 ± 1.82 mg GAE/g and 86.48 ± 0.22 mg QE/g, respectively [14]. The variation in the ethanolic extract might be due to the maturation period, geographical location, and method of extraction. The total phenol content of the methanolic extract from the seed of *L. sativum* at 46 mg GAE/g [40]. The variation might be due to the solvent, method of extraction, and geographical location of the study plant [41]. Furthermore, the total phenolic content of *A. corrorima* seed extracts was comparable to those of *L. sativum* extract. This indicated that the extracts were responsible for the free radical scavenging activity associated with oxidative stability [42,43].

The primary mechanism underlying the antioxidant activity of phenolic compounds is their redox properties, which can be helpful in absorbing and neutralizing free radicals, quenching singlet and triplet oxygen, or dissolving peroxides [14]. The TPC of *A. corrorima* seed extracts demonstrated significantly higher levels than previously reported in the literature. The TPC of the *A. corrorima* hydro distillation extract was 3.98 ± 0.27 mg GAE/g for the seed and 1.32 ± 0.07 mg GAE/g for the pod [17]. The disagreement might be due to the method of extraction, the solvent used, the plant seed harvesting period, the different geographical distribution of the plant, and other environmental factors [23]. The principal antioxidants or free radical scavengers in plants are correlated with phenolic compounds [44]. The study results revealed that the two plant extracts might have strong radical scavenging activity due to their greater phenolic content. The bioactivity of phenolic compounds might be associated with their ability to chelate metals, inhibit lipoxygenase, and scavenge free radicals. Moreover, the TFC of *L. sativum* was 37.63 ± 2.14 mg QE/g [14]. The disagreement might be due to the geographical distribution of the plant, the method of extraction and solvent used [39]. Numerous studies have found that flavonoids found in herbs contribute significantly to their antioxidant effects [45]. Flavonoids are extremely powerful scavengers of most oxidizing compounds, including single oxygen and different free radicals [14,46]. The TFC of the hydro-methanolic extract of *A. corrorima* is 19 ± 0.4 mg QE/g which was lower than the current study result [46]. The phenolic compounds are major secondary metabolites that consist of a large group of biologically active compounds. Due to their redox properties, phenolics act as antioxidants and reducing agents.

The antioxidant activity of the plant seed extract was evaluated using the DPPH, ferric reducing power, phosphor-molybdenum, and hydrogen peroxide scavenging assays. Increase the concentration of plant extract to increase the percentage of free radical inhibition. The antioxidant activity of the *L. sativum* was strongly correlated with the positive control. This might be due to the presence of various phytochemicals such as flavonoids and phenolic compounds [22]. The antioxidant activity of different extracts was found to correlate significantly with their total phenolic content, and that *L. sativum* seeds could be used in food supplement preparations or as a food additive, for caloric gain or to protect against oxidation in nutritional products [14]. The percentage inhibition of the *A. corrorima* ethanolic extract was lower when compared to the *L. sativum* and the positive control. This might be due to the lower concentration of flavonoids and phenolic compounds since those compounds have been reported to scavenge free radicals, superoxide, and hydroxyl radicals by transfer [47].

Plant-derived flavonoids possess antidiarrheal, antimicrobial, antioxidant, and anti-inflammatory properties [38]. Polyphenolic compounds and flavonoids form complexes with bacterial cell walls and exert biological functions [48]. Moreover, the antioxidant capacity can be attributed to the extract's chemical composition and polyphenol content [49]. The radical scavenging activity of the plant extract might be due to the secondary metabolites (phenol, tannin, flavonoid, alkaloids, etc.) responsible for molybdenum ion percentage reduction [12,49]. The naturally occurring amounts of H_2_O_2_ in the air, water, human body, plants, microorganisms, and food are at low concentration levels. It is quickly decomposed into oxygen and water to create hydroxyl radicals that can initiate lipid peroxidation [50]. These indicated that *L. sativum* and *A. corrorima* ethanolic extracts exhibited better H_2_O_2_ scavenging activity, which might be attributed to the presence of various secondary metabolites that could donate electrons to hydrogen peroxide, thereby neutralizing it into H_2_O [40]. In general, increasing the concentration of plant extract increases the percentage free radical inhibition. The ethanolic extract of the *L. sativum* and A. corrorima seed (125 μg/mL) displayed a strong percentage H_2_O_2_ scavenging activity. These indicated that *L. sativum* and *A. corrorima* ethanolic extracts exhibited better H_2_O_2_ scavenging activity, which might be attributed to the presence of phenolic groups that could donate electrons to hydrogen peroxide, thereby neutralizing it into H_2_O [51].

The saponification value represents the number of saponifiable units (acyl groups) per unit weight of oil [52,53]. A high SV indicates that the oil contains a higher proportion of low molecular-weight fatty acids or vice versa [52]. The SV, which is expressed in milligrams of potassium hydroxide, is used to calculate the average molecular weight of oil (mg KOH g^−1^ oil) [53]. The saponification values of deep-frying oil increased with the increase in the frying cycle. However, there was a significant variation observed between the groups on each day of frying. The findings of this study were in close agreement with the previous study report and stated that at the elevated cooking temperature of 350 °C, the SV increased to 250 mg of KOH per 100 g of oil and produce more FFA during frying [54]. Furthermore, the results of these studies are supported by the previous similar study report and state that a high SV results in a high level of short-chain fatty acids and higher glycerol content [55].

The acid value of frying oil rises as the frying cycle lengthens. When compared to the positive and plant antioxidant additive groups, groups I and V showed a significant increase. The highest mean AV of 4.82 mg KOHg^−1^ was recorded on the fifth day of frying [56]. The difference in the AV oil might be due to the type of oil used for frying. The AV of oil generally rises with increased frying times. On the sixth day of frying, a food sample containing a group had a higher AV than the other group. An increase in AV could be attributed to the moisture content of the fried product, which accelerates the hydrolysis of oil. It is known that water can promote the hydrolysis of triacylglycerol to form FFA [57]. The FFA (%) content of the oil was randomly increased with increased frying time. FFA levels were found to rise as the number of frying cycles increased, both for heating and frying. In addition to that, the plant extract-containing group and the positive control group showed a significantly lower FFA (%) content compared to group V. These might be due to the transfer of water from the food sample to the oil, which would accelerate the hydrolysis of triglycerides. The increase in AV and FFA was caused by the cleavage and oxidation of double bonds to form carbonyl compounds, which then oxidized to low-molecular weight fatty acids during frying [58]. The study also found that the plant extract additive group and the positive control significantly inhibited FFA enhancement in frying oil.

Peroxide values represent the primary reaction products of lipid oxidation, which can be measured by their ability to liberate iodine from potassium iodide [57]. PV is the most widely used test for determining the state of oxidation in fats and oils. It also indicates the fat's or oil's rancidity or degree of oxidation, but not stability [57,59]. A carbonyl bond such as aldehyde was generated during the secondary lipid oxidation, and it can react with the anisidine value (0.25% in glacial acetic acid) solution, forming a yellow-colored solution. Significant decrements in the PV of oil were observed after the addition of the plant extract compared to the normal control and the frying oil with food (group V) throughout the study period. This indicated that the plant extract prevent the oxidation of oil up on frying. These might be due to the bioactive secondary metabolite that responsible to prevent oil oxidation. Moreover, the herbal extract with food sample significantly prevents the degradation of oil during excessive frying (6 day). The food sample with herbal extract significantly maintains the degradation of oil. The PV increases during the first 20 frying cycles at 160 °C, and then decreases [60]. The significant variation might be due to the frying cycle and oil type. Based on the amendments made to the Malaysian Food Act 1983 by the Food (Amendment) (No. 3) Regulations 2014, the maximum PV of cooking oil is 10 meq O_2_/kg of oil (Food Act, 1983). The higher the peroxide values, the more oxidized the oil. Additionally, the process of oil breakdown is significantly influenced by the water content or humidity. Moreover, high frying temperatures make peroxide unstable; it quickly breaks down and becomes a dimer and a volatile chemical [61]. Therefore, the greater PV on the fried food containing oil was due to the deterioration and degradation of the oil.

Anisidine analysis is the appropriate method for evaluating secondary lipid oxidation. The *p-AV* in frying oil is an indication of organic peroxides that decompose into secondary products, including alcohols, carboxylic acids, aldehydes, and ketones. The quality of oil can be determined by evaluating the absorbance at 350 nm of the solution [62]. Aldehydes formed during oxidative degradation are secondary decomposition products, and the non-volatile portion of carbonyls remains in the frying oil [63]. The higher *p-AV* of frying oil was revealed throughout the study. This indicated the formation of primary and secondary oxidation products. Similarly, the plant extract additive group significantly decreased the *p-AV* of frying oil compared to the normal control and the food sample (group V). A lower *p-AV* indicates that less rancid oil is produced [61]. However, the plant extract and the positive control group significantly retarded the oxidation of frying oil compared to the food sample and the normal group throughout the study period. The *Pandanus amaryllifolius* leaf extract significantly decreased the level of *p-AV* throughout the study period, which was due to the secondary metabolite in the plant extract [30]. The results of these studies revealed that the thermal degradation of the aldehydes formed at higher temperatures results in a lower accumulation of oil at the higher frying temperature [64]. Moreover, the results of these studies are not in good agreement with the previous study report from Felix A. et al. (1998), which stated that the maximum *p-AV* was reached on the second day of frying for both frying temperatures and then decreased consistently until the end of frying time. The disagreement might be due to oil replenishment in the previous study, different oil types used in the frying cycle, and temperature. Moreover, the TOTOX value of the frying oil was evaluated primary and secondary oxidation of oil. The TOTOX index is a good indicator of the total deterioration of fats and oils. The lower the TOTOX value, the better the frying oil quality [57,63]. Oxidation proceeds very slowly at the initial stage, taking time to reach a rapid increase in oxidation rate. The TOTOX value is a common approach to determining the resistance to oxidative rancidity of edible oils. The TOTOX value after the initial days of frying showed a significant difference between the plant extract-containing group, the normal control, and group V (frying with food) throughout the study period (P < 0.05). The TOTOX value of all the oil was extremely greater than the proposed limit, which is an indication of oil oxidation. The variation might be due to the oil type, the frying condition, and the frying cycle. A similar study was also conducted by *Morienga oliefera* and showed lower *p-AV* and TOTOX values than either soybean oil or palm olein heated at 185 °C for 30 h. Since TOTOX was correlated with PV and *p-AV*, the oil-containing plant extract significantly reduced the TOTOX value compared to the normal control and the food containing frying oil. The TOTOX value was given a more accurate description related to the oxidative conditions of the cooking oil after repeated frying. The lower the TOTOX value, the better the oil quality. The good-quality vegetable oils have TOTOX values of ≤4 [59].

The measurement of total polar compounds is useful in estimating heat misuse in frying oils [5]. Evaluating total polar compounds has been characterized as one of the best indicators of the overall quality of oils, and it provides critical information about the total amount of newly formed compounds having a higher polarity than triacylglycerol [64]. The formation of total polar compounds, which indicates oil deterioration, is strongly related to the primary and secondary oxidation that takes place during frying [65]. The result of this study revealed that the total polar compounds of groups I and V were greater than the standard limit after five days of frying. However, the plant extract additive and the positive control group were less than the standard limit throughout the study period. This indicates that the oil should be avoided after five days of frying, but the plant extract showed a positive effect on the reuse of the oil even after six days of frying. This might be due to the plant antioxidant that prevents the oil from thermal degradation [59]^.^ When the amount of total polar components reaches 25%, oil is considered to be thermally degraded and should be replaced with fresh oil [64,66]. The IV was a direct determination of the unsaturation level of the oil. Iodine was used to halogenate the double bonds in the unsaturated fatty acids. Commonly, frying leads to a reduction in unsaturation, thus indicating a decrease in double bonds. The IV of frying oil in the positive control and the 0.2% w/v *L. sativum* and 0.3% w/v plant extract additive groups were comparable. However, after day 3, there was a significant difference between the 0.3% w/v *A. corrorima* additive and the positive control group. A decrease in the IV throughout the cycles is consistent with the decrease in double bonds as oil becomes oxidized. The oils such as olive, soybean, and sunflower had lower iodine values [67]. However, the addition of plant extract did not appear to reduce the oxidation as the cycle progressed compared to the normal control and the food sample-containing group. As a result, the reduction in the iodine value of the oil up to the sixth frying day was caused by complex physicochemical changes in the oil, which resulted in an unstable characteristic against susceptible oxidative rancidity. These study results were also in line with the previous study report, which stated that the decrease in the IV of the oils after frying shows relatively higher oxidation [2]. Furthermore, the current study is consistent with previous research by Pineda et al. [68], who discovered a decrease in the IV of olive oil, high oleic sunflower oil, and sunflower oil while frying. It might be caused by a decline in the oil samples unsaturation.

The increase in oxidation rate can also be observed in the change of specific absorptivity at 232 and 270 nm, which measures the contents of conjugated dienes (CDs) and conjugated trienes (CTs). The K_232_ is associated with the generation of primary oxidation products (i.e., conjugated dienes), and the K_270_ is used to determine fat oxidation, with parameter values varying depending on oxidation conditions (i.e., conjugated trienes). Conjugated dienes and trienes are a good measure of the primary oxidation of the oil [57]. Double bonds in lipids are changed from non-conjugated to conjugated bonds upon oxidation [69]. The CD and CT levels of frying oil increased with increased frying cycles throughout the study period. The CD of three oil samples increased with a longer frying cycle at 160 °C [68]. The result of this study was also in line with a previous similar report and stated that the CD value of sesame oil increased throughout the frying period [63]. The CD and CT levels of groups I and V were greater than the positive and the plant extract additive groups. However, the *A. corrorima* seed extract additive group showed a lower CD and CT level compared to the *L. sativum* extract additive group. The CD value of sesame oil increased throughout the frying period. In general, the lower CD and CT of the plant extract additive group indicate the potential antioxidant activity of the plant extract, which helps stabilize the oil during repetitive frying. The formation of hydroperoxide from polyunsaturated fatty acids leads to the conjugation of the penta-diene structure. This causes the absorption of UV radiation at 230–234 nm for conjugated dienes. When hydrogen abstraction happens on two active methylenes on C-11 and C-14, it produces two pentadienyl radicals, which result in the production of a mixture of conjugated dienes and trienes [57,63]. High extinction coefficients (K_232_ and K_270_) are an indication of advanced oil deterioration [59]. This leads to an increase in UV absorption at 270 nm attributable to conjugated trienes, apart from 232 nm for CD.

The greatest reductions in pH values were observed in groups I and V. These might be due to the degradation and hydrolysis of oil to form FFAs. The formation of FFAs during thermal treatment is an important dynamic of vegetable oils that may be related to the decrease in pH [70]. The plant extract additive group significantly maintains the reduction of pH compared to the normal control and food control groups. These indicate the oil was stabilized by plant antioxidants. The pH value of frying oil decreases as the frying cycle (days) is increased throughout the entire group. The greater amount of density that was recorded in group V might be due to the formation of high-molecular-weight polymeric compounds upon frying [55]. The density of frying oil that contained food samples was due to the transfer of food samples to the oil, which increased with each frying cycle. The moisture content of oil increases with the length of the frying cycle. This might be due to the mass transfer that occurs during the frying process, which includes water loss, oil absorption, and heat transfer. The presence of water in food and oil speeds up the hydrolysis of the oil and protects it from oxidation during frying. The increase in the moisture content of the oil could be caused by the oil being exposed to food moisture and air humidity from its surroundings, which could aid in rancidity and oxidative stress [35]. The higher moisture content in this group might be due to the accumulation of water from the food sample on the oil and the exposure of the oil to food moisture and air humidity from the environment, which could facilitate rancidity and oxidative stress on the oil [71]. The frying process involves mass transfer, including water loss, oil absorption, and heat transfer [35,71]. The water content in food and oil accelerates the hydrolysis of the oil and also provides protection against oxidation of the oil during frying. Thus, the longer the frying cycle, the longer the oil is exposed to the humidity of the environment. In a nutshell, both plant additive groups significantly inhibited the elevation of moisture content compared to the normal and food sample-containing groups.

The RI is a parameter that is related to molecular weight, fatty acid chain length, degree of unsaturation, and conjugation. To detect adulteration in edible oils, the refractive index can be used as a quality control technique [72]. The RI is affected by the content of saturated and unsaturated fatty acids. Increasing the frying cycle decreases the refractive index value. This indicated that the unsaturated part of the oil was removed and more saturation was formed during frying. The result of this study is not correlated with the previous study report and stated that the RI increase is believed to be related to the high saturated fatty acid content and the non-hydrogenation of palm oil, making it less resistant to heat [55]. The deviation might be due to the reaction conditions and the frying cycle. However, the study was consistent similar study report and illustrated that the RI values decrease as the temperature is increased [73]. This might be due to the formation of Tran's fatty acid upon oxidation, which affects the change in the RI value. Furthermore the *trans*-acids formed during hydrogenation affect refractive index values but not iodine values. There was no statistical variation in the RI value between the groups. However, the plant additive group and the positive control did not significantly prevent the enhancement of the RI value compared to the normal control and the oil with food throughout the study period. The RI value of the plant extract additive group is nearly constant compared to the other groups except the positive control. This indicates that the antioxidant potential of the plant extract inhibits the physicochemical changes of deep frying.

The plant extract and the positive control affected the position of the band, and it showed a shift when the proportion of fatty acids changed. The FTIR spectra of the normal control and the food sample-containing group showed strong OH bands at 3300-3500 cm^−1^, respectively (Fig. 4a and e). These indicated that the triglyceride molecules were degraded and FFA was formed during frying. However, there was no significant variation between the positive control (Fig. 4b) and the plant-treated group. The percentage transmittance of the food sample additive and normal control group were higher, indicating that absorbance was decreasing. This could be attributed to oil hydrolysis and degradation into FFA. Continuous frying of corn and mustard oil samples increases the transmittance and hydrolyzes the triglyceride molecule [74]. The FTIR spectra also showed intense bands in the region of 2950–3000 cm^−1^ that were assigned to sp^3^ carbon stretching, which was for the terminal methyl group of the fatty acid chain. The medium peak at 2800 cm^−1^ was assigned for aliphatic CH_2_ stretching. Carbonyl stretching of the ester functional group (C=O) stretching is assigned to the medium peak at 1600-1700 cm^−1^. However, the peak shift to the greater wave number that was observed in this region in groups I and V might be due to the effect of frying. The strong, intense peaks at 980–1100 cm^−1^ indicate C–O (CH_3_O^−^) stretching of the ester. The medium peaks at 1480 cm^−1^ indicate CH bending of sp^3^ carbon (alkane) [75, 76].

## 5 Conclusions

The quality of the palm oil used in this study was significantly affected by accelerated and deep frying, as revealed by assessing the physicochemical parameters. The plant extract additive group showed a significant improvement in oil quality throughout the study period. The FTIR spectra of frying oil revealed the formation of free fatty acids in the normal control and food sample-containing groups. The positive control and plant extract-treated groups significantly retarded oil degradation and maintained oil quality. Therefore, the optimum concentration of *L. sativum* (0.2% w/v) and *A. corrorima* (0.3% w/v) extract were recommended to the restaurants or street food vendors used as an alternative antioxidant. Moreover, the identification of organic compounds that retard the degradation and oxidation of oil needs to be investigated. Further study on various medicinal plants should be investigated to enhance the oil's stability and investigate the potential substitution of synthetic antioxidants.

## CRediT authorship contribution statement

All authors agreed to submit to the current journal, provided final approval of the version to be published, contributed significantly to the data collection, conception, conceived, designed the experiments, performed the experiments, analysis, and interpretation of data, wrote the paper or critically revised it for important intellectual content, and agreed to be responsible for all aspects of the work.

## Data availability

The optimization parameter for accelerated oxidative were submitted as a supplementary material and the rest of the data available on the corresponding author up on request.

## Declaration of competing interest

The authors declare that they have no known competing financial interests or personal relationships that could have appeared to influence the work reported in this paper.

## References

[1] Shahid M.Z., Saima H., Yasmin A., Nadeem M.T., Imran M., Afzaal M. (2018). Antioxidant capacity of cinnamon extract for palm oil stability. Lipids Health Dis..

[2] Emelike N.J.T., Ujong A.E., Achinewu S.C. (2020). Physicochemical and antioxidant properties of oils used by local fried food vendors in D/Line-Port harcourt, rivers state. Agri. Food Sci. Res..

[3] Esfarjani F., Khoshtinat K., Zargaraan A., Mohammadi-Nasrabadi F., Salmani Y., Saghafi Z., Hosseini H., Bahmaei M. (2019). Evaluating the rancidity and quality of discarded oils in fast food restaurants. Food Sci. Nutr..

[4] Galano A. (2017). Free radicals induced oxidative stress at a molecular level: the current status, challenges and perspectives of computational chemistry based protocols. J. Mex. Chem. Soc..

[5] Phing Y., Ing B., Abas F., Ming O., Wang Y., Arbi I., Mohamed H., Mossad M., Ping C. (2019). Fl uence of carbohydrate- and protein-based foods on the formation of polar lipid fraction during deep-frying. Food Control.

[6] Jaarin K., Kamisah Y. (2012). Repeatedly heated vegetable oils and lipid peroxidation. Lipid Peroxidation.

[7] FAO; WHO (2019). Report of the 26th session of the CODEX committee on fats and oils. Codex Aliment. Comm. Codex Stand. named Veg. oils..

[8] Taghvaei M., Jafari S.M. (2015). Application and stability of natural antioxidants in edible oils in order to substitute synthetic additives. J. Food Sci. Technol..

[9] Banu M., Prasad N., Siddaramaiah (2016). Effect of antioxidant on thermal stability of vegetable oils by using ultrasonic studies. Int. Food Res. J..

[10] Blasi F., Cossignani L. (2020). An overview of natural extracts with antioxidant activity for the improvement of the oxidative stability and shelf life of edible oils. Processes.

[11] Musakhanian J., David J., Masumi R. (2022). Oxidative stability in lipid formulations : a review of the mechanisms , drivers , and inhibitors of oxidation. AAPS PharmSciTech.

[12] Malar J., Chairman K., Singh A.R.J., Vanmathi J.S., Balasubramanian A., Vasanthi K. (2014). Antioxidative activity of different parts of the plant Lepidium sativum linn. Biotech. Reports..

[13] Chatoui K., Talbaoui A., Aneb M., Bakri Y., Harhar H., Tabyaoui M. (2016). Phytochemical screening, antioxidant and antibacterial activity of Lepidium sativum seeds from Morocco. J. Mater. Environ. Sci..

[14] Chatoui K., Harhar H., El Kamli T., Tabyaoui M. (2020). Chemical composition and antioxidant capacity of Lepidium sativum seeds from four regions of Morocco. Evidence-Based complement. Alternative Med..

[15] Wang D., Xiao H., Lyu X., Chen H., Wei F. (2023). Lipid oxidation in food science and nutritional health : a comprehensive review. Oil Crop Sci..

[16] Yahya N.Y., Ngadi N. (2020).

[17] Eyob S., Martinsen B.K., Tsegaye A., Appelgren M., Skrede G. (2008). Antioxidant and antimicrobial activities of extract and essential oil of korarima (Aframomum corrorima (braun) P.C.M. Jansen). Afr. J. Biotechnol..

[18] Hamzah R.U., Jigam A.A., Makun H.A., Egwim E.C. (2013). Antioxidant properties of selected african vegetables, fruits and mushrooms: a review. Mycotoxin Food Saf. Dev. Ctries..

[19] Taylor P., Ghai K., Gupta A.K., Gupta P.K. (2015).

[20] Schum H.K., Essien E.E., Thomas P.S., Oriakhi K., Choudhary M.I. (2017). Characterization and antioxidant activity of volatile constituents from different parts of Aframomum danielli (hook). Medicines.

[21] Zohra F.T. (2015). Extraction of secondary metabolites , phytochemical screening and the analysis of antibacterial activity in stevia rebaudiana. BRAC Univ..

[22] Rao U.S.M., Abdurrazak M., Mohd K.S. (2016). Penyaringan fitokimia, Jumlah asai kandungan flavonoid dan fenolik pelbagai ekstrak pelarut tepal musa paradisiaca. Malaysi. J. Anal. Sci..

[23] Kedir W.M., Dubiwak A.D., Ahmed E.T. (2022). Nephroprotective effect of Asparagus africanus lam. Root extract against gentamicin-induced nephrotoxicity in Swiss albino mice. J. Toxicol..

[24] Mwamatope B., Chikowe I., Tembo D.T., Kamanula J.F., Masumbu F.F.F., Kumwenda F.D. (2023).

[25] Nyero A., Anywar G.U., Malinga G.M. (2023).

[26] Muhammed B.L., Seid M.H., Habte A.T. (2021). Determination of caffeine and hydrogen peroxide antioxidant activity of raw and roasted coffee beans around habru woreda, Ethiopia using UV-vis spectroscopy. Clin. Pharm. Adv. Appl..

[27] Bhatti M.Z., Ali A., Ahmad A., Saeed A., Malik S.A. (2015). Antioxidant and phytochemical analysis of ranunculus arvensis L. Extracts. BMC Res. Notes.

[28] Siddiqui N., Rauf A., Latif A., Mahmood Z. (2017). Spectrophotometric determination of the total phenolic content, spectral and fluorescence study of the herbal unani drug gul-e-zoofa (nepeta bracteata benth). J. Taibah Univ. Med. Sci..

[29] Woldegiorgis A.Z., Abate D., Haki G.D., Ziegler G.R. (2014). Antioxidant property of edible mushrooms collected from Ethiopia. Food Chem..

[30] Mohd F., Mohamed S., Aini N., Ismail R. (2008). Antioxidative properties of pandanus amaryllifolius leaf extracts in accelerated oxidation and deep. Frying Stud..

[31] Kariminejad M., Naimabadi A., Morshedi A., Id T.M., Shokuhi A., Bordbar M. (2023).

[32] Aziz A.A., Elias S.M., Sabran M.R. (2018). Repeatedly heating cooking oil among food premise operators in bukit mertajam , pulau pinang and determination of peroxide in cooking oil. Malaysi. J. Med. Heal. Sci..

[33] Durmaz F., Talpur M.Y. (2015). Oxidation on the stability of canola oil blended with stinging nettle oil at frying temperature. Int. J. Food Prop..

[34] Tadesse N., Reta N., Beyero N. (2017). Level of saturation and anti-oxidant value of heat and spice treated animal butter. Food Publ. Health.

[35] Jurid L.S., Zubairi S.I., Kasim Z.M., Kadir I.A.A. (2020). The effect of repetitive frying on physicochemical properties of refined, bleached and deodorized Malaysian tenera palm olein during deep-fat frying. Arab. J. Chem..

[36] David F., Mattn P.Y. (2011).

[37] Fesseha Y. (2020). Phytochemical screening of some selected home garden plants in amhara region, north gondar, gondar. Ijesc.

[38] Abeysinghe D.T., Kumara K.A.H., Kaushalya K.A.D., Chandrika U.G., Alwis D.D.D.H. (2021). Phytochemical screening, total polyphenol, flavonoid content, in vitro antioxidant and antibacterial activities of Sri Lankan varieties of murraya koenigii and micromelum minutum leaves. Heliyon.

[39] Tarchoune I., Sgherri C., Eddouzi J., Zinnai A., Quartacci M.F., Zarrouk M. (2019). Olive leaf addition increases olive oil nutraceutical properties. Molecules.

[40] Ahamad R., Mujeeb M., Anwar F., Ahmad A. (2015). Phytochemical analysis and evaluation of anti-oxidant activity of methanolic extract of Lepidium sativum L. Seeds. Der Pharm. Lett..

[41] Karazhiyan H., Razavi S.M.A., Phillips G.O., Fang Y., Al-Assaf S., Nishinari K., Farhoosh R. (2009). Rheological properties of Lepidium sativum seed extract as a function of concentration, temperature and time. Food Hydrocolloids.

[42] Liu S., Huang H. (2015). Assessments of antioxidant effect of black tea extract and its rationals by erythrocyte haemolysis assay , plasma oxidation assay and cellular antioxidant activity (CAA) assay. J. Funct.Foods.

[43] More G.K., Makola R.T. (2020).

[44] Saeed N., Khan M.R., Shabbir M. (2012). Antioxidant activity , total phenolic and total flavonoid contents of whole plant extracts torilis leptophylla L. BMC Compl. Alternative Med..

[45] Bitis L., Sen A., Ozsoy N., Birteksoz-Tan S., Kultur S., Melikoglu G. (2017). Flavonoids and biological activities of various extracts from rosa sempervirens leaves. Biotechnol. Biotechnol. Equip..

[46] Dessalegn E., Bultosa G., Haki G.D., Chen F., Rupasinghe H.P.V. (2022). Antioxidant and cytotoxicity to liver cancer HepG2 cells in vitro of korarima (aframomumcorrorima (braun) P.C.M. Jansen) seed extracts. Int. J. Food Prop..

[47] Gangwar M., Gautam M.K., Sharma A.K., Tripathi Y.B., Goel R.K., Nath G. (2014). Antioxidant capacity and radical scavenging effect of polyphenol rich Mallotus philippenensis fruit extract on human erythrocytes: an in vitro study. Sci. World J..

[48] Mo Y., Cheng F., Yang Z., Shang X., Liang J. (2021).

[49] Rahman M.M., Islam M.B., Biswas M., Khurshid Alam A.H.M. (2015). In Vitro antioxidant and free radical scavenging activity of different parts of tabebuia pallida growing in Bangladesh. BMC Res. Notes.

[50] J Mbah C., Orabueze I., H Okorie N. (2019). Antioxidants properties of natural and synthetic chemical compounds: therapeutic effects on biological system. Acta Sci. Pharm. Sci..

[51] Yazdizadeh Shotorbani N., Jamei R., Heidari R. (2013). Antioxidant activities of two sweet pepper capsicum annuum L. Varieties phenolic extracts and the effects of thermal treatment. Avicenna J. Phytomed..

[52] Ismail S.A.A., Ali R.F.M. (2015). Physico-chemical properties of biodiesel manufactured from waste frying oil using domestic adsorbents. Sci. Technol. Adv. Mater..

[53] Ismail S.A.E.A., Ali R.F.M. (2015). Physico-chemical properties of biodiesel manufactured from waste frying oil using domestic adsorbents. Sci. Technol. Adv. Mater..

[54] Zubair M., Baig A., Summra S.H., Nazar M.F., Nadeem M., Jabeen K., Hassan M.B., Rashid U., Tan C.P., Tan T.B. (2021). Heating effect on quality characteristics of selected canola cooking oils. Physiochem. Sta. Anal..

[55] Kpata-Konan N.E., Yao N.B., Coulibaly K.J., Konan K.F. (2020). Determination of physico-chemical indices of frying oils used by attieké-fish sellers in daloa (Mid-West of CôTe d'Ivoire). Food Nutr. Sci..

[56] Idun-Acquah N., Obeng G.Y., Mensah E. (2016). Repetitive use of vegetable cooking oil and effects on physico-chemical properties-case of frying with redfish (Lutjanus fulgens). Sci. Technol..

[57] Nayak P.K., Dash U., Rayaguru K., Krishnan K.R. (2016). Physio-chemical changes during repeated frying of cooked oil: a review. J. Food Biochem..

[58] Sharoba A.M., Ramadan M.F. (2012).

[59] Flores M., Meyer L., Orellana S., Saravia C., Galdames C., Perez-Camino M.C. (2018). Quality of lipid fractions in deep-fried foods from street vendors in Chile. J. Food Qual..

[60] Velasco J., Marmesat S., Carmen Dobarganes M. (2008). Chemistry of frying. Adv. Deep. Fry. Foods.

[61] Marmesat S., Mancha M., Ruiz-Méndez M.V., Dobarganes M.C. (2005). Performance of sunflower oil with high levels of oleic and palmitic acids during industrial frying of almonds, peanuts, and sunflower seeds. JAOCS, J. Am. Oil Chem. Soc..

[62] Turgut Dunford N. (2015).

[63] Borjian Borojeni M., Goli S.A.H., Gharachourloo M. (2016). Effect of roasted sesame oil on qualitative properties of frying oil during deep-fat frying. J. Agric. Sci. Technol..

[64] Sayyad R. (2017). Effects of deep-fat frying process on the oil quality during French fries preparation. J. Food Sci. Technol..

[65] Debnath S., Rastogi N.K., Gopala Krishna A.G., Lokesh B.R. (2012). Effect of frying cycles on physical, chemical and heat transfer quality of rice bran oil during deep-fat frying of poori: an Indian traditional fried food. Food Bioprod. Process..

[66] Ahmad Tarmizi A.H., Niranjan K., Gordon M. (2013). Physico-chemical changes occurring in oil when atmospheric frying is combined with post-frying vacuum application. Food Chem..

[67] Javidipour I., Erinç H., Baştürk A., Tekin A. (2017). Oxidative changes in hazelnut, olive, soybean, and sunflower oils during microwave heating. Int. J. Food Prop..

[68] Martínez-Pineda M., Ferrer-Mairal A., Vercet A., Yagüe C. (2011). Physicochemical characterization of changes in different vegetable oils (olive and sunflower) under several frying conditions. CyTA - J. Food.

[69] Rafati A., Tahvildari K., Nozari M. (2019). Production of biodiesel by electrolysis method from waste cooking oil using heterogeneous MgO-NaOH nano catalyst. Energy Sources, Part A Recover. Util. Environ. Eff..

[70] Ganatra V.J., Mahapatra A. (2011).

[71] Negash Y.A., Amare D.E., Bitew B.D., Dagne H. (2019). Assessment of quality of edible vegetable oils accessed in gondar city, northwest Ethiopia. BMC Res. Notes.

[72] Khan M., Rahman A., Chowdhury U.K., Officer S.S. (2016).

[73] Dijkstra A. (2015).

[74] Zahir E., Saeed R., Hameed M.A. (2017). yousuf, A. Study of physicochemical properties of edible oil and evaluation of frying oil quality by fourier transform-infrared (FT-IR) spectroscopy. Arab. J. Chem..

[75] Schönemann A., Edwards H.G.M. (2011). Raman and FTIR microspectroscopic study of the alteration of Chinese tung oil and related drying oils during ageing. Anal. Bioanal. Chem..

[76] Banerjee S., Kumar S., Mandal A., Naiya T.K. (2017). Design of novel chemical solvent for treatment of waxy crude. Int. J. Oil Gas Coal Technol..

