# Supplementary material for: Antioxidant activity of selected plants extract for palm oil stability via accelerated and deep frying study
Source: Heliyon. 2023 Jul 7;9(7):e17980. doi: 10.1016/j.heliyon.2023.e17980 (PMC10362148; doi:10.1016/j.heliyon.2023.e17980)
Supplement: Multimedia component 1 [file mmc1.docx]

**Supplementary material**

- **Calibration curve of the total phenolic and flavonoid contents**

**Figure 1A.** Calibration curve of standard gallic acid for determination of total phenol.

**Figure 2A.** Calibration curve of standard quercetin for the determination of total flavonoid.

## ***Accelerated oxidative study***

To evaluate the effect of frying during accelerated oxidative study, different concentrations of plant extract (0.1-0.4%) were investigated and compared with the positive control BHA for 0 to 72h.

### **The Acid value (a), saponification value (b), Iodine value (c) and peroxide value (d) of frying oil during accelerated oxidative study**


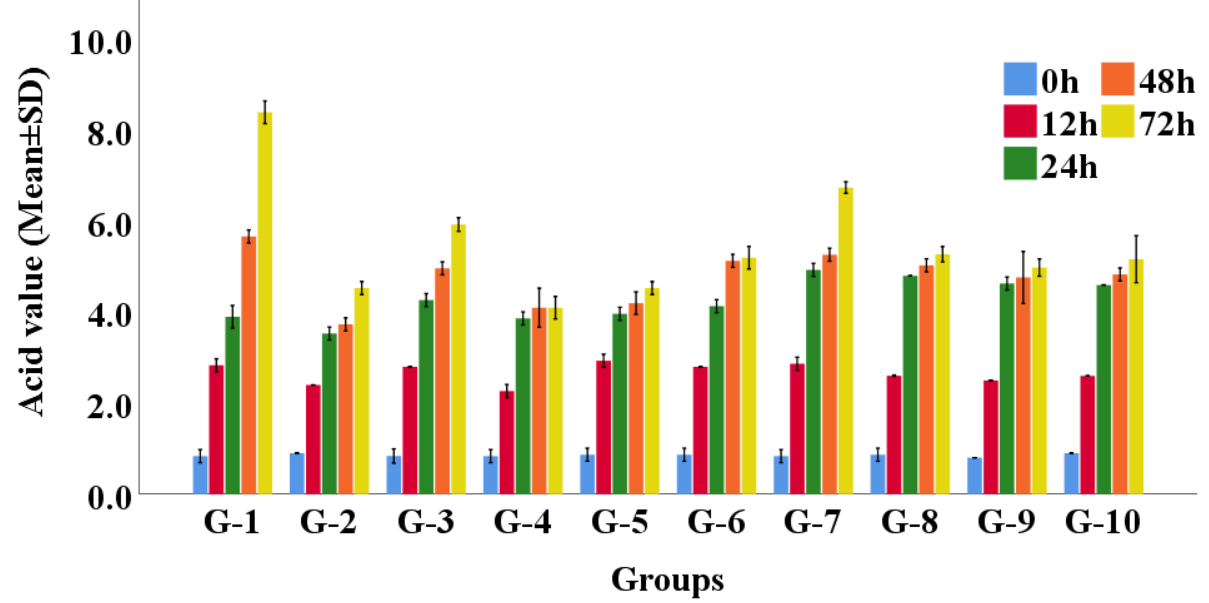

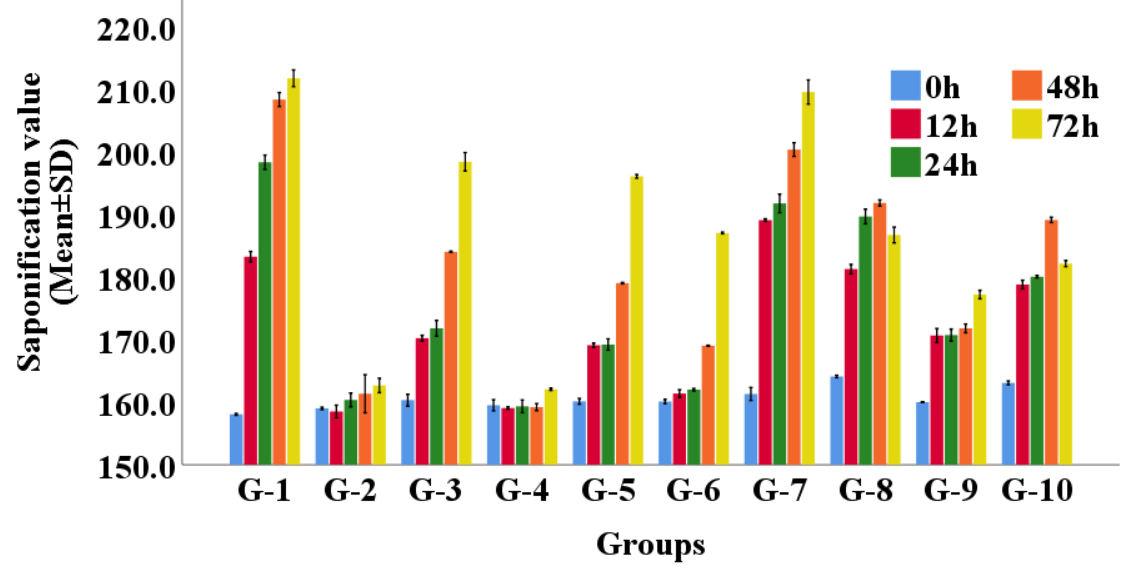


(**b**)

(**a**)


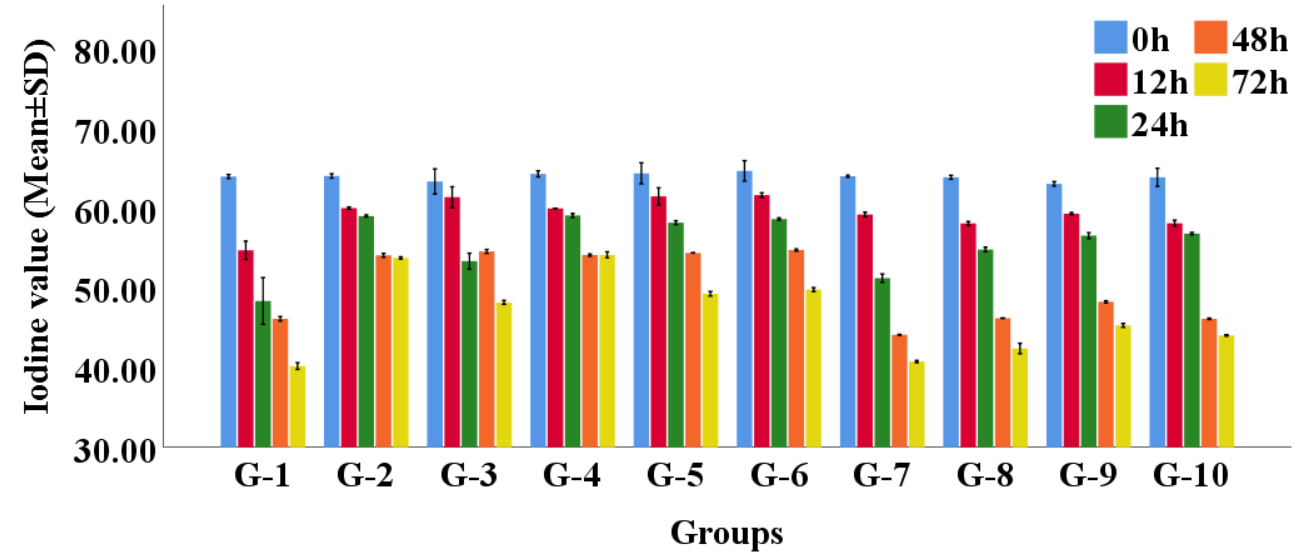

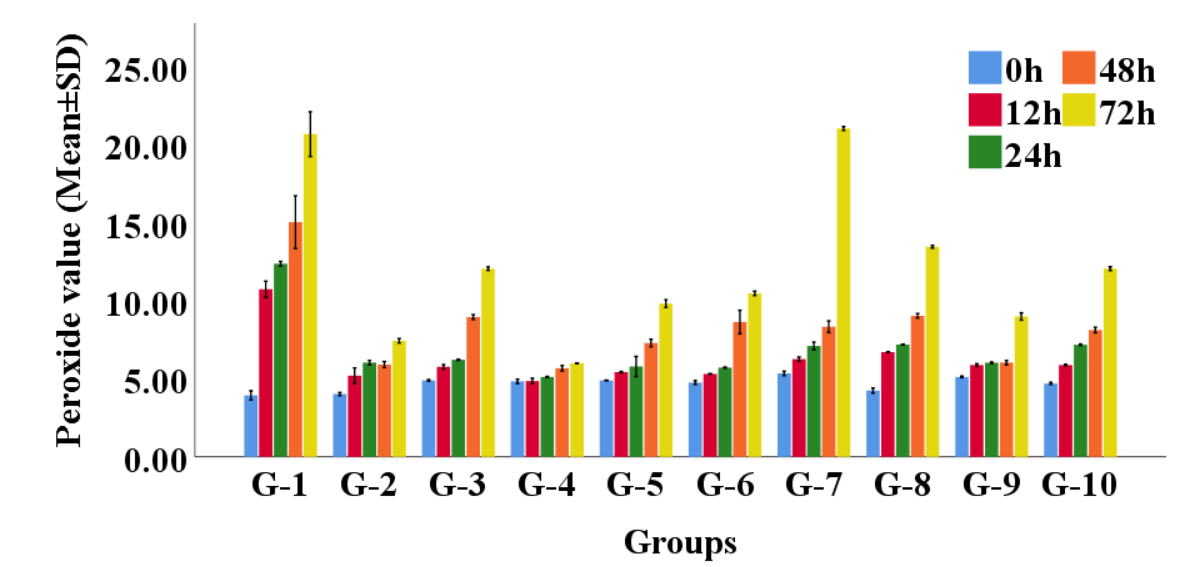


(**d**)

(**c**)

**Figure 3A.** The Acid value saponification value, Iodine value and peroxide value of frying oil during accelerated oxidative study.

### **The Total polar compound (a) and Conjugate diene and Conjugate trienes (b) of frying oil during accelerated oxidative study.**

(**b**)

(**a**)


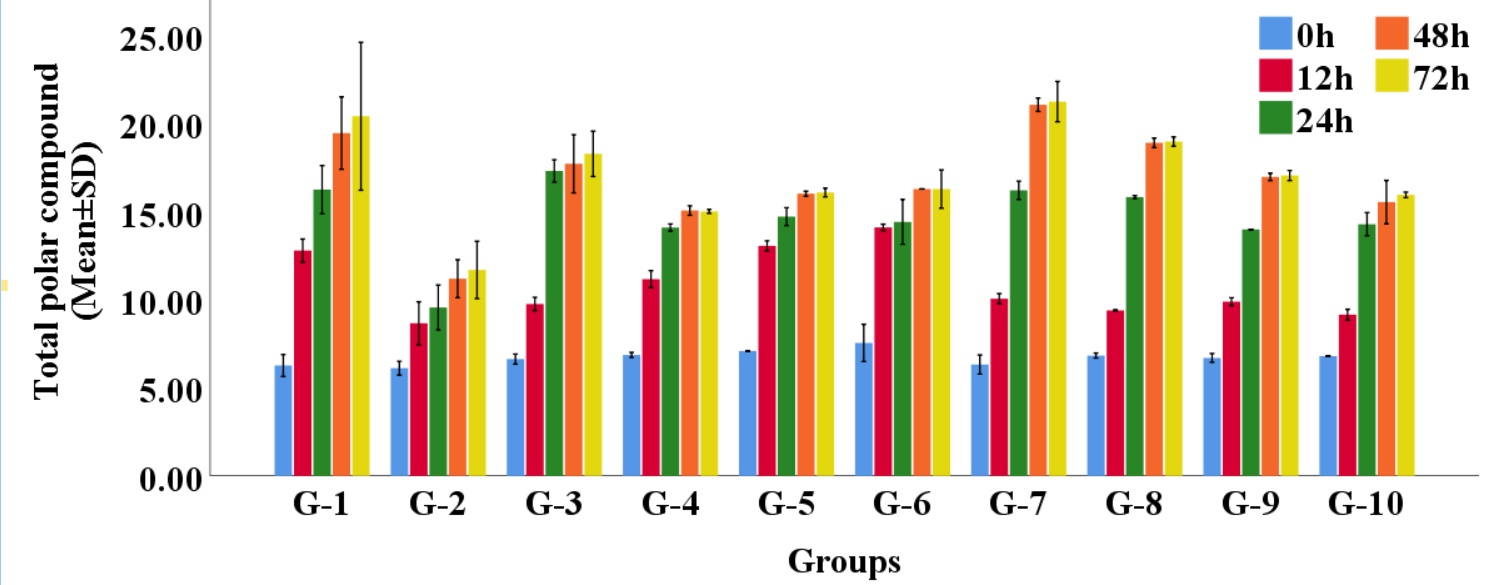

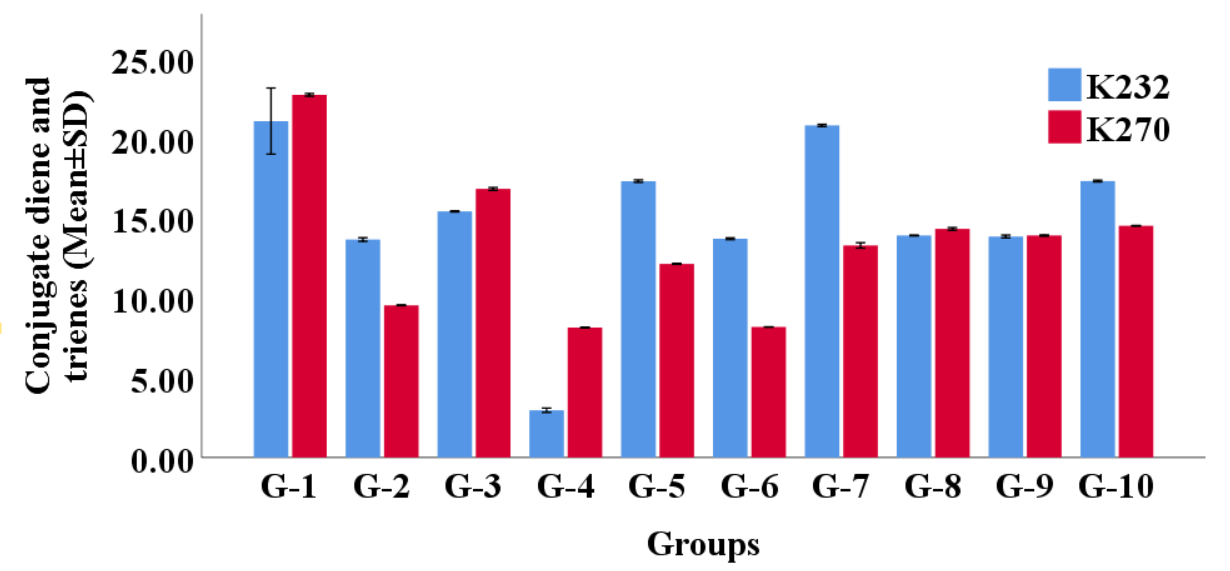


**Figure 4A.** Total polar compound, Conjugate diene and Conjugate trienes of flying oil during accelerated oxidative study.
